# Zein and Trimethyl Chitosan-Based Core–Shell Nanoparticles for Quercetin Oral Delivery to Enhance Absorption by Paracellular Pathway in Obesity Mice

**DOI:** 10.34133/bmr.0193

**Published:** 2025-04-28

**Authors:** Zijian Dai, Wanting Yin, Jiahao Li, Lingjun Ma, Fang Chen, Qun Shen, Xiaosong Hu, Yong Xue, Junfu Ji

**Affiliations:** National Engineering and Technology Research Center for Fruits and Vegetables, College of Food Science and Nutritional Engineering, China Agricultural University, Beijing 100083, P.R. China.

## Abstract

Quercetin as a flavonoid polyphenol in nature has shown great anti-obesity effects. Due to its poor stability in chemical structure and low intestinal absorption, the in vivo bioavailability of quercetin is considered to be the main challenge for applications. To achieve the oral quercetin administration, chitosan was successfully trimethylated (TMC) to coat the quercetin-loaded zein nanoparticles (Zein-Q), which were designed as the core–shell structure for enhancing the intestinal absorption in this study. TMC-Zein-Q was demonstrated to protect quercetin from degradation and showed the sustained-release effect in an in vitro drug release experiment. The nanoparticles were found to reversibly open tight junctions between intestinal epithelial cells and help to increase quercetin uptake via the paracellular pathway in Caco-2 cells. In addition, the delivery system also showed stronger intestinal permeability and mucoadhesion in vivo, which improved the bioavailability of quercetin in cellular and animal experiments. After 10 weeks of intervention, TMC-Zein-Q could effectively suppress weight gain, improve serum lipid levels, and ameliorate hepatic steatosis and glucose tolerance in high-fat diet (HFD) mice by mediating the AMPK pathway. Consequently, this work successfully constructed TMC-Zein-Q for oral quercetin delivery, providing a novel and feasible strategy for the treatment of obesity via the oral route.

## Introduction

Quercetin is a typical flavonoid ubiquitously present in fruits and vegetables with a series of outstanding bioactivities, including antioxidant, anti-inflammatory, and anti-obesity effects [1]. Over 650 million adults worldwide are obese, and about 1.67 billion people will have health problems due to obesity or being overweight by 2025, seriously threatening human health [2]. Accumulating evidence suggests that quercetin could ameliorate obesity by inhibiting lipogenesis, promoting lipolysis, regulating insulin sensitivity, and alleviating inflammation and oxidative stress [3,4]. However, due to its low aqueous solubility and poor stability in the gastrointestinal tract, quercetin generally exhibits low oral absorption [1]. In addition, the absorbed quercetin undergoes extensive phase II metabolism in intestinal cells [5], and most of the related metabolites are excreted back to the intestinal lumen [6], limiting the utilization of quercetin as a nutraceutical in food and supplement products. The results of human experiments showed that the absorption rate of quercetin after oral administration was only 3% to 17% [7]. The total plasma concentration of quercetin cannot reach the threshold of effective therapeutic concentration after eating quercetin-rich foods [8,9]. Among these influencing factors involved in the oral bioavailability of quercetin, the low intestinal absorption is the most important [10], and therefore, there is an urgent need to improve intestinal absorption of quercetin after oral administration.

Various delivery systems were designed to efficiently protect quercetin in the harsh gastrointestinal tract against degradation, improving its bioavailability and therapeutic effects [2]. Among these strategies, nanoparticles have been proven to be an effective strategy to improve the absorption and metabolism of bioactive compounds. They could embed bioactive compounds driven by self-assembly and release them in a controlled manner at specific sites in the body [11]. The transcellular and paracellular pathways are 2 common ways for nanoparticles to improve the absorption of bioactive compounds. The paracellular pathway can effectively avoid the efflux of bioactive compounds that undergo extensive phase II metabolism in intestinal cells, thereby increasing their bioavailability [12]. Several strategies such as poly(amidoamine) dendrimers and inorganic nanoparticles (gold and silicon) have shown improved intestinal absorption after modification via paracellular pathway [13,14]. However, the major challenges that involve the clinical translation of these nanoparticles as efficient oral delivery carriers are their toxicity and biocompatibility [13].

Food-derived materials are commonly used as nano-embedding materials due to their advantages of nontoxicity, bioavailability, nonimmunogenicity, biocompatibility, and tunability, and some of them have been approved by the Food and Drug Administration (FDA) for clinical applications [15,16]. Zein is the main storage protein of corn and has been approved for use by the FDA [17]. There is a high proportion of hydrophobic amino acids in its structure, which is effective in encapsulating and loading various hydrophobic compounds. In addition, zein could spontaneously form nanoparticles with anti-gastric enzymes, making it an ideal candidate for an oral delivery vehicle [17]. However, zein nanoparticles could not avoid the efflux of quercetin, showing a limited improvement in absorption. Therefore, further methods are needed to improve the intestinal absorption of quercetin. Some cationic polymers have been shown to interact with the negatively charged intestinal epithelial cells, changing the absorption model of bioactive compounds by activating the paracellular pathway [18]. TMC is a typical cationic material modified by chitosan, which is derived from chitin, and the safety and biodegradability of TMC are well established [12]. Although chitosan is widely used in various delivery systems, its poor water solubility and stability in neutral and alkaline environments limits its further application [19]. In contrast, TMC overcomes these shortcomings of traditional chitosan. It exhibits better solubility and permeability under neutral pH conditions, enabling more efficient promotion of quercetin absorption [20]. Furthermore, the cationic properties of TMC enhance its interaction with intestinal epithelial cells, thereby improving drug adhesion in the intestine and promoting absorption across the intestinal barrier [18]. These advantages make TMC a highly promising candidate for oral drug delivery systems, particularly in applications related to anti-obesity and antioxidant effects, where it has demonstrated higher bioavailability. Therefore, TMC as a shell coated with zein nanoparticles for oral delivery of quercetin may be a potential strategy to improve quercetin absorption via the paracellular pathway.

In this study, a novel core–shell delivery system by using zein and TMC was constructed, which simultaneously exhibited intestinal permeability and mucoadhesion capacities. Following characterization of the physicochemical properties and stability, the intestinal absorption of TMC-Zein-Q was comprehensively evaluated by in vitro digestion, ex vivo imaging, and assessment of pharmacological effects. The possible mechanisms of enhanced bioavailability of quercetin were also explored based on cellular and animal experiments. Furthermore, the subsequent weight loss of nanoparticles in vivo and the potential signaling pathway were also investigated. In conclusion, our findings suggested that TMC-Zein-Q could largely increase the bioavailability of quercetin by paracellular pathway and showed great potential as an oral delivery system for obesity intervention.

## Materials and Methods

### Materials

Quercetin (98% purity) and zein were purchased from Shanghai Yuanye Biotechnology Co. Ltd. (Shanghai, China). Chitosan (50 kDa, 95% deacetylation degree) and *N*-methyl-2-pyrrolidone were purchased from Macklin Biochemical Co. Ltd. (Shanghai, China). Pepsin and trypsin were purchased from Sigma-Aldrich Chemical Co. (St. Louis, MO, USA). Methyl iodide was purchased from Titan Scientific Co. Ltd. (Shanghai, China). Fetal bovine serum (FBS) was purchased from Thermo Fisher Scientific Inc. (Waltham, MA, USA). Dulbecco’s modified Eagle’s medium (DMEM), nonessential amino acids, l-glutamine, penicillin, streptomycin, Hanks’ balanced salt solution (HBSS), sodium azide, chlorpromazine, indomethacin, and colchicine were obtained from Solarbio Science & Technology Co. Ltd. (Beijing, China).

### Synthesis and characterization of TMC

TMC was synthesized as previously described with some modifications [21]. Briefly, 10 g of chitosan, 24 g of sodium iodide, and 55 ml of 15% NaOH were dissolved in 400 ml of *N*-methyl-2-pyrrolidone with stirring at 60 °C for 30 min. Then, under a refluxing condition, 57.5 ml of methyl iodide was added into the above mixture and reacted in the dark for 90 min. The obtained solution was precipitated by adding ethanol, centrifuged to collect the sediment, and washed with ether for 2 times. The recovered material was dissolved in 200 ml of 10% (w/v) NaCl solution followed by dialysis for 3 d. The final solid TMC sample was obtained by freeze-drying.

Fourier-transform infrared (FTIR) analysis of chitosan and TMC was performed by an FTIR spectrometer (PerkinElmer, Massachusetts, USA). The sample was mixed with potassium bromide powder and pressed into transparent sheets. Proton nuclear magnetic resonance (^1^H-NMR) spectra of TMC were recorded by using an NMR spectrometer (JEOL, Akishima-shi, Japan) using D_2_O as solvent. The degree of quaternization of TMC is calculated according to the following formula:

$$\text{Degree of quaternization}=\frac{\int \mathrm{TM}}{\left(\int H\right)/9}\times 100\%$$ (1)

where ∫TM is the peak area integral corresponding to the trimethylamine group, and ∫H is the peak area integral corresponding to the hydrogen atom bound by C1 on the glucopyranose ring.

### Synthesis and characterization of the TMC-Zein-Q core–shell nanoparticles

To determine the most appropriate wall materials, nanoparticles with different ratios of zein to TMC (8:1, 4:1, 2:1, 1:1, and 1:2) were prepared. TMC was dissolved in 150 ml of ultrapure water and adjusted to pH 4.0, and 600 mg of zein (Yuanye, Shanghai, China) was dissolved in an 80% ethanol solution. The TMC solution was mixed by a high shear rate of 3,000 rpm, where the zein ethanol solution was slowly added. The TMC-Zein nanoparticles were freeze-dried after removing ethanol by rotary evaporation. The particle size and ζ potential of nanoparticles were measured by a particle size analyzer (Malvern, Marvin, UK), and the turbidity was measured by a turbidimeter (Beckman Coulter, California, USA).

The fabrication of TMC-Zein-Q was set as follows: Zein and quercetin were dissolved together in an 80% ethanol solution according to rations of 40:1, 20:1, 10:1, 5:1, and 2.5:1, respectively. Then, the solution was slowly added into TMC solution (pH 4) under high-speed shear. TMC-Zein-Q was freeze-dried and obtained following rotary evaporation and centrifugation to remove the unencapsulated quercetin. Zein-Q was also prepared as the control.

The fluorescence spectra of zein, Zein-Q, TMC-Zein-Q, and TMC-Zein were detected by fluorescence spectrometry (Hitachi, Tokyo, Japan) with the samples dissolved under the protein concentration of 28 μg/ml. The excitation wavelength was 280 nm, the emission spectrum was 300 to 500 nm, and the slit was 10 nm. The FTIR analysis of zein, TMC, quercetin, Zein-Q, TMC-Zein-Q, and TMC-Zein was performed with the same method mentioned above. TMC-Zein-Q was diluted by 20 times and dried on a carbon-coated copper mesh to be observed by a transmission electron microscope.

Encapsulation efficiency (EE) and drug loading capacity (DLC) were determined by measuring the amount of unencapsulated quercetin and total quercetin in the nanoparticles. TMC-Zein-Q (20 mg) was added in 5 ml of anhydrous methanol, and the suspension was vortexed and centrifuged at 10,000*g* for 5 min. The content of quercetin in the supernatant was considered as the amount of unencapsulated quercetin. In addition, 20 mg of nanoparticles was dissolved in 0.5 ml of ultrapure water, followed by the addition of 4.5 ml of methanol and ultrasonic treatment for 5 min to fully destroy the nanoparticle structure. The solution was also centrifuged, and the quercetin content in the supernatant was considered as the total quercetin content. Quercetin was quantified by high-performance liquid chromatography (HPLC) (Shimadzu, Tokyo, Japan) with a C18 column (4.6 × 150 mm, 5 μm) and a ultraviolet (UV) detection wavelength at 370 nm. A mixture of methanol and water (70:30, v/v) was used as the mobile phase. The volumes of injection were 20 μl with a flow rate of 1.0 ml/min. EE and DLC were calculated using the following formula:

$$\begin{array}{c} \mathrm{EE}=\frac{\text{Weight of total quercetin}-\text{Weight of unencapsulated quercetin}\;}{\text{Weight of total quercetindsd}\;}\\ \;\times 100\% \end{array}$$ (2)

$$\begin{array}{c} \mathrm{DLC}=\frac{\text{Weight of total quercetin}-\text{Weight of unencapsulated quercetin}\;}{\text{Weight of nanocarriers}}\\ \;\times 100\% \end{array}$$ (3)

### In vitro stability of the TMC-Zein-Q core–shell nanoparticles

Freshly prepared TMC-Zein-Q solutions were adjusted to pH 3.0 to 8.0 and NaCl concentration of 0 to 500 mM to evaluate the pH stability and the ionic strength stability of the nanoparticles, respectively. The samples were kept at room temperature for 3 h, and then the particle size was measured.

### In vitro drug release study

The method used was slightly modified according to a previous report [22]. Briefly, simulated gastric fluid (pepsin 2,000 U/ml) and simulated intestinal fluid (trypsin 100 U/ml) were prepared in advance. Nanoparticles were dissolved in ultrapure water at a protein concentration of 0.2 mM. The solution was then mixed with an equal volume of simulated gastric fluid, and the pH was adjusted to 3.0 immediately. The digestive solution was incubated in a 37 °C water bath with a shaking rate of 110 rpm for 2 h. Then, the pH of obtained chyme was adjusted to 7.0 and mixed with an equal volume of simulated intestinal fluid for 2 h under the same conditions. During the digestion process, 0.2-ml samples were taken from the digestive solution every 30 min and freeze-dried. The obtained powder was added to 0.2 ml of anhydrous methanol and vortexed for 30 s to extract quercetin released from the nanoparticles. The quercetin content in the supernatant was quantified, and 50 mg/ml of free quercetin suspension was used as the control group. The release rate of quercetin was calculated according to the following formula:

$$\begin{array}{c} \text{Release rate}=\frac{\text{Content of quercetin in digestive solution}}{\text{Total content of quercetin in digestive solution}}\\ \;\times 100\% \end{array}$$ (4)

### Cell culture

Caco-2 cells were purchased from Peking Union Medical College and cultured in a high-glucose DMEM with 10% (v/v) FBS, 1% (v/v) nonessential amino acids, 1% (v/v) l-glutamine, 100 U/ml penicillin, and 100 μg/ml streptomycin in a 37 °C incubator with 5% CO_2_ and 95% humidity. The growth medium was changed every other day. For transport assays, the cultured cells (passage numbers 40 to 45) were seeded onto the Transwell permeable supports (Corning, Cambridge, USA) at 1 × 10^5^ cells/well [23]. Monolayers were fed on both sides every 2 d and cultured for 21 d before use. When the transepithelial electrical resistance (TEER) reached 700 to 800 Ω cm^2^, it indicated the formation of monolayers.

### In vitro cytotoxicity assay

The effects of free quercetin, Zein-Q, and TMC-Zein-Q on the activity of Caco-2 cells at different concentrations were evaluated by the methylthiazolyldiphenyl-tetrazolium bromide (MTT) Assay Kit (Beyotime, Shanghai, China). Caco-2 cells were seeded at a density of 5 × 10^3^ cells per well in 96-well plates and cultured for 24 h. Cells were then incubated with free quercetin, Zein-Q, and TMC-Zein-Q at 0.2 to 1 mg/ml equivalent weight of quercetin, respectively. The control group applied HBSS to incubate cells, while the blank group only used HBSS without cells. After treatment for 12 and 24 h, the cell viability was tested by MTT assay and calculated according to the following formula:

$$\begin{array}{c} \text{Cell viability}=\frac{\text{The sample absorbance value}-\text{The blank absorbance value}}{\text{The control absorbance value}-\text{The blank absorbance value}}\\ \;\times 100\% \end{array}$$ (5)

### In vitro cellular uptake study

Caco-2 cells were seeded in 12-well plates (1 × 10^5^ cells/well) and cultured to 75% to 80% confluency. Free quercetin, Zein-Q, and TMC-Zein-Q (0.8 mg/ml equivalent weight of quercetin) were used to incubate the cells for 2 h at 37 °C. Then, the cells were washed twice with phosphate-buffered saline (PBS). The cells were ultrasonic treated and detected by HPLC to quantify the cellular uptake of quercetin. To determine if the nanoparticle uptake was energy dependent, the Caco-2 cells were incubated with the samples at 4 °C and pre-incubated with 100 mM sodium azide for 30 min, separately. To determine the mechanism of cell uptake, the cells were pre-incubated (30 min) with endocytosis inhibitors (10 μg/ml chlorpromazine, 10 μg/ml indomethacin, or 10 μg/ml colchicine) for clathrin-, caveolae-, and macropinocytosis-dependent cell uptake, respectively [24].

### In vitro transepithelial permeability study

Transepithelial permeabilities of free quercetin, Zein-Q, and TMC-Zein-Q were determined by using Caco-2 cell monolayers. The donor solution in the apical chamber of the Transwell plate was replaced with 0.5 ml of free quercetin, Zein-Q, or TMC-Zein-Q (0.8 mg/ml equivalent weight of quercetin). After 0.5, 1, 1.5, and 2 h of incubation, 100-μl samples were collected from the basolateral chamber. The concentration of quercetin in the samples was detected by HPLC. The apparent permeability coefficient (*P*_app_) was calculated according to the following formula:

$$P_{\mathrm{app}}=\frac{dQ}{dt}\times \frac{1}{A\times C}$$ (6)

where d*Q*/d*t* is the permeation amount of quercetin per second (ng/s), *A* is the diffusion area of the cell monolayer (cm^2^), and *C* is the initial concentration of quercetin in the apical chamber (ng/cm^3^).

During the incubation, the TEER values of the cell monolayers were measured by Millicell-Electrical Resistance System (Millipore Corporation, MA, USA). After incubation with the samples for 2 h, the cell monolayers were washed twice by PBS and subsequently cultured in fresh cell culture media. The TEER values were recorded at predetermined time intervals in the next 24 h. The percentage of TEER values relative to the initial level was calculated.

### Immunofluorescence imaging of occludin protein

The status of tight junction between the Caco-2 cells after the incubation with nanoparticles was visualized by the immunofluorescent staining of occludin protein. Nanoparticles were prepared by replacing quercetin with Nile Red (NR) (Aladdin, Shanghai, China). The cell monolayers were incubated with free NR, Zein-NR, or TMC-Zein-NR for 2 h. Then, the cells were fixed in 4 % paraformaldehyde, permeabilized in 0.5 % Triton-X PBS (Solarbio, Beijing, China), and treated with 5% donkey serum (Solarbio, Beijing, China) for 1 h to block nonspecific binding. Subsequently, the cells were treated with occludin goat polyclonal immunoglobulin G (IgG) (Santa Cruz, California, USA) at a dose of 1:1,000 at 4 °C overnight. Following the washing steps, the cells were incubated with Alexa Fluor 488-labeled donkey anti-goat IgG (1:100 in PBS) (Abcam, Cambridgeshire, UK) for 1 h. The stained cells were visualized by confocal laser scanning microscopy (CLSM) (Hitachi, Tokyo, Japan).

### Animals studies

Male C57BL/6J mice (6 weeks old) were purchased from Beijing Vital River Laboratory Animal Technology Co. Ltd. (Beijing, China). They were reared in the specific pathogen-free (SPF) animal laboratory under controlled temperature (23 ± 2 °C) and relative humidity (55 ± 5%) with a 12-h cycle of light–dark. After 1 week of acclimatization, the mice were used for subsequent studies. All the procedures were approved by the Animal Care Committee of China Agricultural University (AW72110202-4) and performed in strict accordance with the guidelines of the National Research Council Guidelines.

### Ex vivo intestinal permeability study

Male C57BL/6J mice were sacrificed, and their fresh duodenum, jejunum, and ileum were collected and cut into small segments (3 cm). One end of the intestinal segment was tightened with medical cotton thread, while 0.3 ml of free quercetin, Zein-Q, or TMC-Zein-Q samples (0.8 mg/ml equivalent weight of quercetin) was injected from the other end and then closed. The selected length of the intestinal segment was 3 cm. The intestinal segments were placed in 3 ml of 37 °C Krebs–Ringer buffer (Solarbio, Beijing, China). After incubation for 2 h, 100 μl of each sample was collected from the solution. The concentration of quercetin was determined by HPLC for the calculation of *P*_app_.

### In vivo intestinal permeability and mucoadhesion study

For intestinal permeability study, male C57BL/6J mice were given free NR, Zein-NR, or TMC-Zein-NR by gavage. After 2 h, the mice were sacrificed, and the small intestine tissues were collected, which were embedded in optimal cutting temperature compounds (Sakura, Osaka, Japan). The thick sections (8 μm) were cut and fixed with 4% paraformaldehyde. For the observation of CLSM, the nuclei and intestinal mucus were stained with 4′,6-diamidino-2-phenylindole (DAPI) (Thermo, Massachusetts, USA) and Alexa Fluor 488 WGA (Thermo, Massachusetts, USA), respectively. For intestinal mucoadhesion study, male C57BL/6J mice were given free Cy7, Zein-Cy7, or TMC-Zein-Cy7 by gavage. The mice were sacrificed at 2, 4, 8, and 12 h, and the complete digestive systems were taken and observed under an animal in vivo fluorescence imager (Spectral Instruments, Arizona, USA).

### Pharmacodynamic study in mice

Male C57BL/6J mice were fed by free quercetin, Zein-Q, or TMC-Zein-Q by gavage at a dose of 50 mg/kg body weight. The mice were sacrificed at 0.25, 0.5, 1, 2, 4, 6, 8, and 12 h after administration (*n* = 4 per point), and the blood was isolated at 4 °C (3,500 rpm, 15 min). The serum samples (100 μl) were added with 20 μl of enzyme solution (800 U/ml β-glucuronidase and 200 U/ml sulfate esterase) (Sigma, Missouri, USA), vortexed for 30 s, and then treated in 37 °C water bath for 1 h. Subsequently, 20 μl of HCl solution (2.5 M), 10 μl of ferulic acid (Sigma, Missouri, USA), and 1 ml of ethyl acetate were added, followed by vortexing for 5 min and centrifuging at 14,000 rpm for 5 min. The supernatant was dried with nitrogen and then redissolved in 100 μl of acetonitrile for HPLC analysis, using a C18 column (4.6 × 150 mm, 5 μm) and a UV detector (detection wavelength = 366 nm). A mixture of acetonitrile and 0.2% phosphoric acid (30:70, v/v) was used as the mobile phase. The temperature of column was set at 30 °C, and the volumes of injection were adjusted at 20 μl with a flow rate of 1.0 ml/min.

### Anti-obesity study in mice

Forty male C57BL/6J mice were randomly divided into 5 groups (*n* = 8 per group): (a) control group: the mice were fed a normal chow diet (D12450J, 10% energy derived from fat, 3.85 total kcal/g, Research Diets Inc., New Brunswick, NJ, USA); (b) high-fat diet (HFD) group: the mice were fed an HFD diet (D12492, 60% energy derived from fat, 5.24 total kcal/g, Research Diets Inc., New Jersey, USA); (c) HFD with free quercetin group (HFD + free Q): the mice were fed an HFD and free quercetin (quercetin content: 50 mg/kg, once every day, intragastrically); (d) HFD with Zein-Q group (HFD + Zein-Q): the mice were fed an HFD and Zein-Q (quercetin content: 50 mg/kg, once every day, intragastrically); (e) HFD with TMC-Zein-Q group (HFD + TMC-Zein-Q): the mice were fed an HFD and TMC-Zein-Q (quercetin content: 50 mg/kg, once every day, intragastrically). All groups were fed for 10 weeks, food intake was evaluated twice per week, and body weight was recorded once per week. The energy intake was calculated by multiplying the grams of diet by the energy (kcal/g) in the diet. At the end of the experiment, the mice were anesthetized with pentobarbital sodium and sacrificed. The blood samples were taken from the orbital vascular plexus after 12-h fasting, and the serum was isolated at 4 °C (3,500 rpm, 15 min) and then stored at −80 °C until required. The liver, epididymal fat (epi-WAT), retroperitoneal fat (retro-WAT), and perirenal fat (per-WAT) were collected and weighed. Part of these tissues was fixed in 4% paraformaldehyde for histological analysis, while the remainder were stored at −80 °C for further experimental analysis.

### Serum lipid analysis

The levels of triglyceride (TG), total cholesterol (TC), low-density lipoprotein cholesterol (LDL-c), and high-density lipoprotein cholesterol (HDL-c) in serum were measured by commercially available kits according to the instructions of the manufacturer (Jiancheng, Nanjing, China).

### Oral glucose tolerance test

The oral glucose tolerance test (OGTT) was carried out at 9 weeks after a 12-h overnight fast. All mice were given 20% glucose solution (2 g/kg body weight) orally, and venous blood was collected from the tails. The blood glucose levels were measured by using a glucose meter (Byer, Shanghai, China) at 0, 15, 30, 60, 90, and 120 min, and the area under the curve (AUC) of glucose was calculated to reflect the glucose tolerance in this study.

### Histological analysis

The liver and epi-WAT tissues were dehydrated using a series of ethanol solutions after being fixed in 4% paraformaldehyde (v/v) for more than 24 h. Then, the tissues were treated with xylene, immersed in paraffin at 65 °C, and embedded in paraffin wax. The sections of 4 μm were cut by a microtome and dried at 60 °C in an oven. After that, the slices were dewaxed with xylene and dyed with hematoxylin and eosin (H&E). The slices were sealed with neutral gum, dried, and viewed under a microscope (Olympus Corporation, Tokyo, Japan). Additionally, the liver tissues were also performed by Oil Red O staining. Briefly, tissues were dehydrated at 4 °C in a 15% sucrose solution and then transferred to a 30% sucrose solution at 4 °C. The dehydrated tissues were slightly dried with filter paper and embedded in optimal cutting temperature compounds. The freezing microtome (Thermo, Massachusetts, USA) was used to cut the sections (8 to 10 μm), which were dyed with oil red. Finally, the slices were sealed with a glycerol gelatin sealing agent and viewed under a microscope.

### Quantitative real-time PCR

Target gene expressions were assessed using quantitative real-time PCR (RT-qPCR) on epi-WAT mRNA, which was isolated by a Trizol reagent (Invitrogen, Waltham, MA, USA). The quantity and purity of RNA were assessed by absorbance at 260 and 280 nm, and reverse transcribed with a PrimeScript RT Reagent Kit with gDNA Eraser (Takara, Osaka, Japan). Template cDNA (1.5 μl) was mixed with 0.5 μl of primers, 5 μl of TB Green Premix Ex Taq II (Takara, Osaka, Japan), and 3 μl of ribonuclease (RNase)-free water. PCRs were performed in triplicates on a LightCycler 480 Real-Time PCR system according to the following program: initial denaturation at 95 °C for 10 min, 35 PCR cycles at 95 °C for 10 s, 60 °C for 30 s, 72 °C for 15 s, followed by a melting curve. The used primers are listed in Table S1. Relative quantification normalized against glyceraldehyde-3-phosphate dehydrogenase (GAPDH) gene expression was calculated by the comparative 2^–∆∆CT^ method with 6 biological replicates.

### Statistical analysis

All experimental data are presented as mean ± standard error of mean (SEM). Comparisons among groups were performed by one-way analysis of variance (ANOVA) followed by the Tukey test (SPSS v20.0, Illinois, USA) and graphed by GraphPad Prism (GraphPad Software, California, USA). *P* < 0.05 was considered as statistically significant.

## Results and Discussion

### Design and characterization of TMC-Zein-Q core–shell nanoparticles

**Fig. 1.**
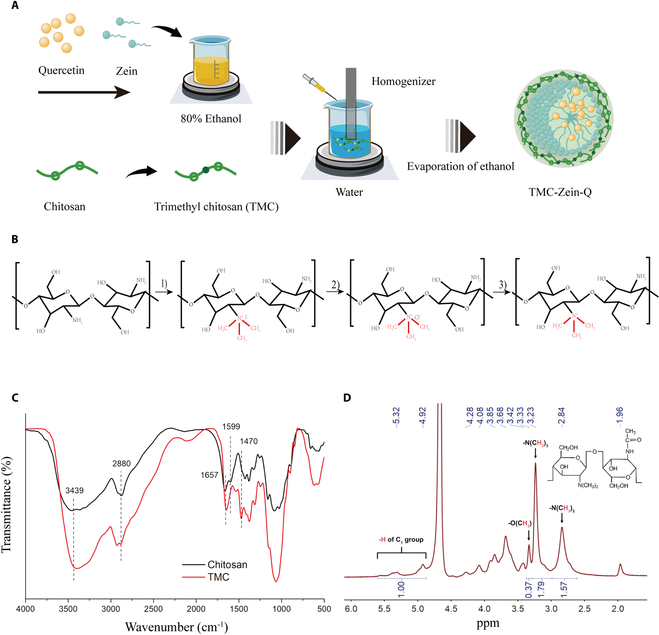
Design and characterization of TMC. (A) Schematic representation of the TMC-Zein loaded with quercetin. Chitosan was trimethylated to obtain TMC, and quercetin was loaded into the hydrophobic core of zein. Zein-Q was subsequently coated by TMC to synthesize TMC-Zein-Q with great intestinal permeability and mucoadhesion properties. (B) Synthesis route of TMC. 1), 2), and 3) represent the reagents and conditions at different stages of the reaction: 1) adding CH_3_I, NaOH, NaI, *N*-methyl-2-pyrrolidone; 2) adding 10% NaCl; 3) dialysis with deionized water. (C) FTIR spectra of TMC and chitosan. The appearance of a new peak at 1,470 cm^−1^ in the FTIR spectrum confirmed the existence of N-CH_3_ in the prepared TMC. The absorption peak at 1,599 cm^−1^ corresponding to the hydrogen of the NH_2_ group in chitosan was partially replaced in TMC, also confirming the formation of TMC. (D) ^1^H-NMR spectrum of TMC. In addition to the trimethyl group, the ^1^H-NMR spectrum of TMC also contained a dimethyl group and an O-methyl group, confirming the formation of TMC.

In the present study, a TMC-Zein nanoparticle with oral absorption enhancement was designed as a carrier to improve the bioavailability of quercetin. To improve the bioavailability of quercetin, food-derived chitosan was trimethylated to obtain TMC shell with good mucoadhesive and intestinal epithelium-penetrating properties. Meanwhile, quercetin was loaded into the hydrophobic core of zein and subsequently combined with TMC to synthesize the TMC-Zein-Q core–shell nanoparticles (Fig. 1A). TMC was prepared according to Fig. 1B, and the chemical structure of TMC was verified by FTIR and ^1^H-NMR (Fig. 1C and D). The presence of N-CH_3_ in TMC was confirmed by the appearance of a new peak at 1,470 cm^−1^ in FTIR spectroscopy. Additionally, the absorption peak at 1,599 cm^−1^ corresponding to the hydrogen of the NH_2_ group in chitosan was partially replaced in TMC, resulting in a decrease in absorption, which also confirmed the formation of TMC (Fig. 1C). The modification efficiency of TMC was analyzed by ^1^H-NMR. As shown in Fig. 1D, in addition to the trimethyl group, the prepared TMC also contained a dimethyl group and an o-methyl group in its structure, and the degree of quaternization of the prepared TMC was 19.98%.

**Fig. 2.**
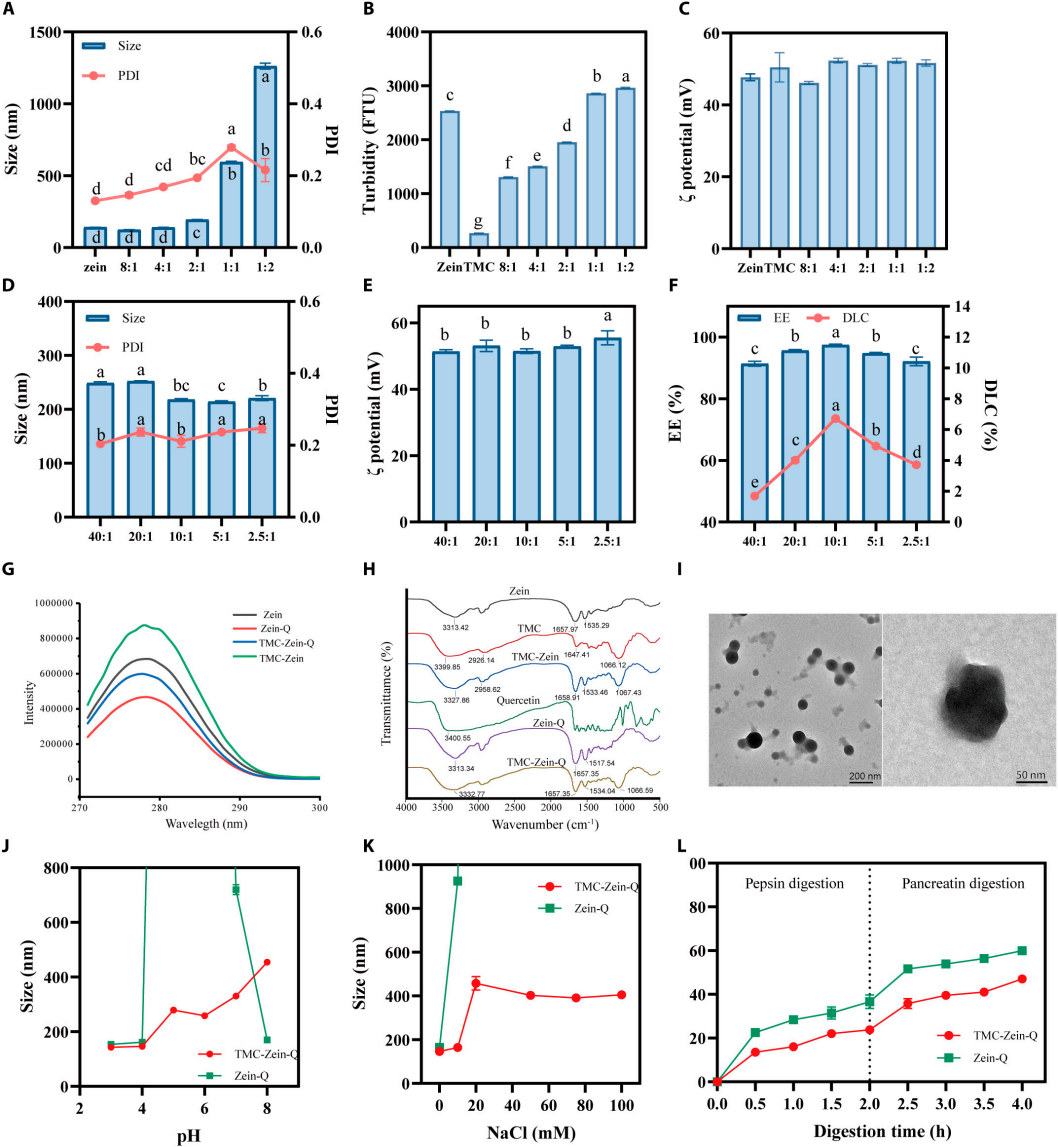
Characterization, stability, and in vitro drug release of TMC-Zein nanoparticles. (A) Particle size, PDI, (B) turbidity, and (C) ζ potential of different ratios of zein to TMC nanoparticles. (D) Particle size, PDI, (E) ζ potential, and (F) EE and DLC of TMC-Zein-Q. (G) Florescence spectrum and (H) FTIR spectra of quercetin-loaded nanoparticles. (I) Transmission electron microscopy images of TMC-Zein-Q (J) Effect of pH and (K) NaCl concentration on the stability of Zein-Q and TMC-Zein-Q. (L) Quercetin release profiles from Zein-Q and TMC-Zein-Q during *in vitro* simulated digestion process. The data are expressed as mean ± SEM (*n* = 3). Different lowercase letters (a, b, c, d) indicate statistically significant differences between groups (*P* < 0.05). Groups sharing at least one common letter are not significantly different from each other, while groups with completely different letters exhibit significant differences. FTU, formazine turbidity units.

In order to determine the optimal ratio of the wall materials, TMC-Zein nanoparticles were prepared according to the ratio of zein:TMC = 8:1, 4:1, 2:1, 1:1, and 1:2, respectively. As shown in Fig. 2A, the particle size of zein nanoparticles was 140.30 ± 0.56 nm, and the polydispersity index (PDI) was 0.13 ± 0.01. After the addition of TMC, the particle size of nanoparticles did not increase significantly, and the PDI was less than 0.2 at the ratios of 8:1, 4:1, and 2:1, indicating that the TMC-Zein particles remained small size and the distribution was uniform. However, when the ratio of Zein/TMC rose to 1:1 and 1:2, the particle size increased sharply, indicating that the addition of excessive TMC led to the adhesion and aggregation of nanoparticles. This point was also demonstrated by the higher turbidity (Fig. 2B). The increase in the proportion of positively charged TMC did not significantly change the surface potential of the nanoparticles (Fig. 2C). Usually, the larger proportion of polysaccharides was beneficial for improving the EE of nanoparticles, while the smaller particle was helpful for taking advantage of their high specific surface area to interact with the corresponding sites in the body. Therefore, based on the above results, zein/TMC at a ratio of 2:1 was selected for subsequent studies.

Furthermore, to achieve the best EE and DLC, the nanoparticles with zein and quercetin at ratios of 40:1, 20:1, 10:1, 5:1, and 2.5:1 were prepared. The particle size, PDI, ζ potential, EE, and DLC of the obtained nanoparticles were shown in Fig. 2D to F. The particle sizes of different nanoparticles loaded with quercetin were all around 200 to 250 nm, and the PDI was around 0.2, indicating that the addition of quercetin had little effect on the particle size and uniformity of TMC-Zein nanoparticles. The ζ potential values of all nanoparticles were almost the same as those without quercetin encapsulation (51.1 ± 0.7 mV). The EE of all nanoparticles was higher than 90%, indicating that Zein-TMC nanoparticles could effectively embed quercetin. However, the DLC of nanoparticles prepared under the condition of zein/quercetin (10:1) could reach 6.71 ± 0.07%, which was significantly higher than those of other samples. Moreover, the results of fluorescence spectroscopy showed that zein exhibited a strong fluorescence emission peak at 278 nm under the excitation of 280 nm (Fig. 2G). When loading quercetin, the endogenous fluorescence intensity of both Zein and TMC-Zein nanoparticles decreased significantly, indicating that quercetin was successfully bound to the wall materials after encapsulation. The occurrence of fluorescence quenching proved that quercetin could be effectively loaded into nanoparticles, which further verified that TMC-Zein-Q had a high EE. Therefore, the ratio of zein/quercetin (10:1) was selected to prepare TMC-Zein-Q for the subsequent experiments.

The absorption peaks at 3,313.42 and 3,399.85 cm^−1^ (-OH stretching vibration) of zein and TMC were shifted in the sample of TMC-Zein (Fig. 2H). These results showed a hydrogen bond between zein and TMC, which may be caused by the amide group of zein and the hydroxyl of TMC [25]. The characteristic peaks at 1,657.97 and 1,535.29 cm^−1^ (C=O stretching and N–H bending) in the zein spectrum, as well as the characteristic peaks at 2,926.14, 1,647.41, and 1,066.12 cm^−1^ (C–H stretching and tilting, C=O stretching, and C–O–C stretching) in the TMC spectrum, all shifted in the TMC-Zein nanoparticle spectrum. These results demonstrated that there was a hydrophobic interaction between zein and TMC, which may be generated by the nonpolar amino acid residues in zein and the acetyl group of TMC [26]. In addition, the original C=O stretching and N–H bending characteristic peaks of zein in Zein-Q spectrum shifted, revealing a hydrophobic interaction between zein and quercetin. Based on the above results, it was speculated that zein, TMC, and quercetin form nanoparticles through hydrophobic interactions and hydrogen bonds.

Furthermore, the morphological features of TMC-Zein-Q were observed by transmission electron microscopy. Spherical zein particles coated by transparent TMC with a core–shell structure could be seen in TMC-Zein-Q (Fig. 2I). According to the results of FTIR analysis, it was speculated that zein combined with quercetin to form a spherical core spontaneously, while TMC was able to coat the outside of zein particles to create a hydrophilic shell. In conclusion, the produced TMC-Zein-Q had a uniform size with the core–shell structure as well as exhibited the high EE and DLC.

### Stability and in vitro drug release of the TMC-Zein-Q core–shell nanoparticles

In the human gastrointestinal tract, there were harsh environments including severe pH changes, high salt concentrations, and numerous proteases [27]. Nanoparticles are generally used to protect encapsulated quercetin from degradation during gastrointestinal digestion [28]. Therefore, the stability of nanoparticles is believed to be a prerequisite to exert the related functionalities in vivo. The stability of Zein-Q was impaired at pH 5.0 to 6.0, as reflected by the significantly increased mean particle size (Fig. 2J). TMC-Zein-Q reversed this effect, indicating that TMC significantly improved the pH stability of nanoparticles. However, the particle size of TMC-Zein-Q increased gradually at pH 5.0 to 8.0, which may be related to the swelling characteristics of TMC under alkaline conditions [29]. In addition, with the increase of pH, the electrostatic repulsion between positively charged TMC molecules weakens the interaction between molecules, and the balance between TMC and Zein changes, resulting in matrix loosening and volume expansion. Zein-Q was very sensitive to the increase of ionic strength, with particle size increasing to 921.0 ± 33.7 nm at only 10 mM concentration of NaCl (Fig. 2K). In contrast, TMC-Zein-Q had higher ionic strength stability. In conclusion, TMC-Zein-Q achieved better quercetin protection under severe pH changes and high ionic strength conditions due to the shell structure of TMC.

In order to better evaluate the digestion characteristics of nanoparticles in the gastrointestinal tract, the release of quercetin from TMC-Zein-Q was carried out by in vitro simulated digestion using gastric and intestinal fluids. During the 2-h gastric digestion stage, the release amount of quercetin in TMC-Zein-Q was only 23.78 ± 0.78 %, which was significantly lower than that of Zein-Q showing 36.66 ± 5.35 % (Fig. 2L). High ionic strength and strong gastric acids were considered as the main reasons to cause serious aggregation and sedimentation of Zein-Q in the stomach. This aggregation could limit the digestion of simulated gastric fluid and showed a certain sustained release of quercetin [30]. However, when TMC shell was applied, it acted as an effective physical barrier to reduce the enzymatic hydrolysis of zein, which could further delay the release of quercetin by maintaining the core–shell structure [31]. This kind of effect was more obvious during intestinal digestion. Similarly, the release amount of quercetin in TMC-Zein-Q was only 47.07 ± 1.21% after 4-h digestion, which was still dramatically lower than that of Zein-Q. Therefore, it can be concluded that TMC-Zein core–shell structures showed an excellent sustained-release effect of quercetin, which could effectively protect quercetin from degradation and be used as an ideal delivery system for the next cellular and animal experiments.

### Cytotoxicity and cellular uptake of TMC-Zein-Q core–shell nanoparticles

**Fig. 3.**
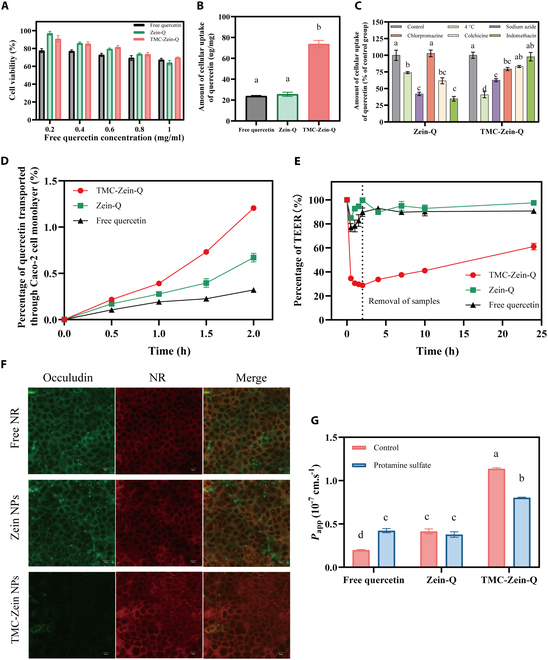
Cytotoxicity and cellular uptake of TMC-Zein-Q. (A) Effects of free quercetin, Zein-Q, and TMC-Zein-Q incubation for 24 h on Caco-2 cell viability. (B) Cellular uptake of free quercetin, Zein-Q, and TMC-Zein-Q by Caco-2 cells. The amount of cellular uptake is expressed as the amount of quercetin internalized per cellular protein. (C) Cellular uptake of quercetin by Caco-2 cells after incubation with Zein-Q or TMC-Zein-Q under different conditions. (D) Percentage of quercetin transported through Caco-2 cell monolayers after treatment with free quercetin, Zein-Q, or TMC-Zein-Q. (E) Effect of free quercetin, Zein-Q, or TMC-Zein-Q on TEER values of Caco-2 monolayer. (F) Immunofluorescence imaging of occludin in Caco-2 cell monolayer after treatment with free NR, Zein-NR, or TMC-Zein-NR for 2 h. (G) Effect of protamine sulfate on the *P*_app_ value of the free quercetin, Zein-Q, or TMC-Zein-Q after 2 h of incubation with Caco-2 cell monolayers. The data are expressed as mean ± SEM (*n* = 3 to 4). Different lowercase letters (a, b, c, d) indicate statistically significant differences between groups (*P* < 0.05). Groups sharing at least one common letter are not significantly different from each other, while groups with completely different letters exhibit significant differences.

Excellent biosafety was a prerequisite for the medical translation of nanoparticles to the clinic [32]. In our study, the results of cytotoxicity showed that the cell activity exhibited a negative correlation with quercetin dose, possibly due to the anticancer effects of quercetin, which could be toxic to cancer cells by activating apoptosis [33]. Interestingly, both Zein-Q and TMC-Zein-Q increased the cell viability compared with the free quercetin group (Fig. 3A), indicating great biocompatibility in Caco-2 cells.

Then, the research evaluated the cellular uptake amounts of nanoparticles. TMC-Zein-Q-treated Caco-2 cells significantly increased the uptake of quercetin, which was apparently due to the enhancement of nanoparticle endocytosis induced by the TMC shell (Fig. 3B). Then, the mechanism of nanoparticle uptake by Caco-2 cells was investigated with different inhibitors. The uptake of quercetin by Caco-2 cells in Zein-Q and TMC-Zein-Q groups was significantly reduced under the conditions of 4 °C and with the addition of sodium azide (Fig. 3C), as both conditions could block active transport processes [18]. It demonstrated that the internalization of the 2 carriers was an active transport that required the participation of energy. Interestingly, chlorpromazine, an inhibitor of clathrin-dependent cellular uptake, significantly decreased the cellular uptake of TMC-Zein-Q instead of Zein-Q. As the inhibitor of caveolin- and macropinocytosis-dependent cell uptake (clathrin-independent endocytosis), indomethacin and colchicine remarkably reduced the cellular uptake of Zein-Q, but no effect on TMC- Zein-Q. These results revealed that clathrin-dependent endocytosis was involved in the cellular uptake of TMC-Zein-Q and clathrin-independent endocytosis was related to the cellular uptake of Zein-Q. It is commonly believed that the cationic properties of chitosan enabled chitosan and its derivative to interact with negatively charged cell membranes, thereby affecting cellular uptake behavior [34]. This point was consistent with a previous study that found that positively charged particles were strictly subject to clathrin-dependent endocytosis [35]. Furthermore, clathrin-dependent endocytosis is the most common route for nanoparticle uptake in nonspecialized mammalian cells, and its endocytosis process is faster than caveolin-dependent endocytosis [36]. Therefore, we speculated that the modification of nanoparticles by TMC changed the original way of nanoparticles entering cells, which may lead to the higher uptake of TMC-Zein-Q by Caco-2 cells [37].

### Transport of TMC-Zein-Q core–shell nanoparticles by paracellular pathway

The paracellular pathway was evaluated using the Caco-2 cell monolayer model. During the 2-h permeability study, the permeation amount of quercetin in TMC-Zein-Q was always higher than those of free quercetin and Zein-Q (Fig. 3D). After 2 h, the permeation amount and *P*_app_ value of quercetin in TMC-Zein-Q were 3.76- and 5.7-fold higher than those of free quercetin, and 1.8- and 2.78-fold higher than those of Zein-Q, respectively. It showed that TMC significantly enhanced the permeability of quercetin in Caco-2 cell monolayers. Moreover, TMC-Zein-Q led to a dramatic reduction in the TEER values of the cell layer (Fig. 3E). After removal of the incubated TMC-Zein-Q, a gradual increase in the TEER value was observed, while this observation was not found in the Zein-Q and free quercetin groups. These results indicated that TMC-Zein-Q might reversibly open the tight junctions between Caco-2 cells, thereby enhancing the permeability of quercetin [18]. Combined with the great biosafety of TMC-Zein-Q, this destruction of the tight junctions between Caco-2 cells was not caused by cytotoxicity.

Occludin protein was the major protein component in tight junctions [38]. In order to identify the effects of TMC-Zein on the tight junctions between Caco-2 cells, the distribution of occludin was observed by CLSM after treating Zein-NR and TMC-Zein-NR nanoparticles on the Caco-2 monolayers. For the free NR and Zein-NR-treated groups, the fluorescence signal of occludin protein in the cell layers showed a complete ring, proving that the cell layer was highly intact (Fig. 3F). Conversely, the occludin fluorescence signal of the TMC-Zein-NR-treated group was significantly weaker, suggesting that the expression of occludin protein was down-regulated compared with other groups. It was implied by the decrease in the TEER value and the down-regulation of occludin expression that the tight junctions were opened by the TMC-Zein nanoparticles. In addition, negligible NR signals were observed in the cells treated with free NR and Zein-NR, indicating that NR given in the above forms hardly entered the cells. Strikingly, the TMC-Zein-NR group exhibited a strong intracellular NR fluorescence signal, revealing that the cellular uptake of nanoparticles was greatly enhanced by TMC-Zein.

Based on the cationic properties of chitosan, it was speculated that TMC may bind to the cell membrane of epithelial cells through electrostatic interactions, thereby affecting the tight junctions between cells [12]. Therefore, positively charged protamine sulfate was added to the sample solution to interfere with the cationic properties of TMC [39]. The result showed that the *P*_app_ value of TMC-Zein-Q was significantly reduced in the presence of protamine sulfate. However, this effect was not found in the Zein-Q-treated group, indicating that the positive charge of TMC had an important influence on the cell uptake of TMC-Zein-Q (Fig. 3G). The *P*_app_ value of free quercetin was also increased, which may be due to the fact that protamine was able to penetrate cells and enter the nucleus, while part of quercetin bound to protamine and entered cells together with it, resulting in increased permeability [40]. Collectively, TMC could interact with epithelial cells through electrostatic adsorption, leading to the reversible opening of intercellular tight junctions, which ultimately enabled quercetin to be transported through a paracellular pathway.

### Intestinal permeability and mucoadhesion of TMC-Zein-Q core–shell nanoparticles

**Fig. 4.**
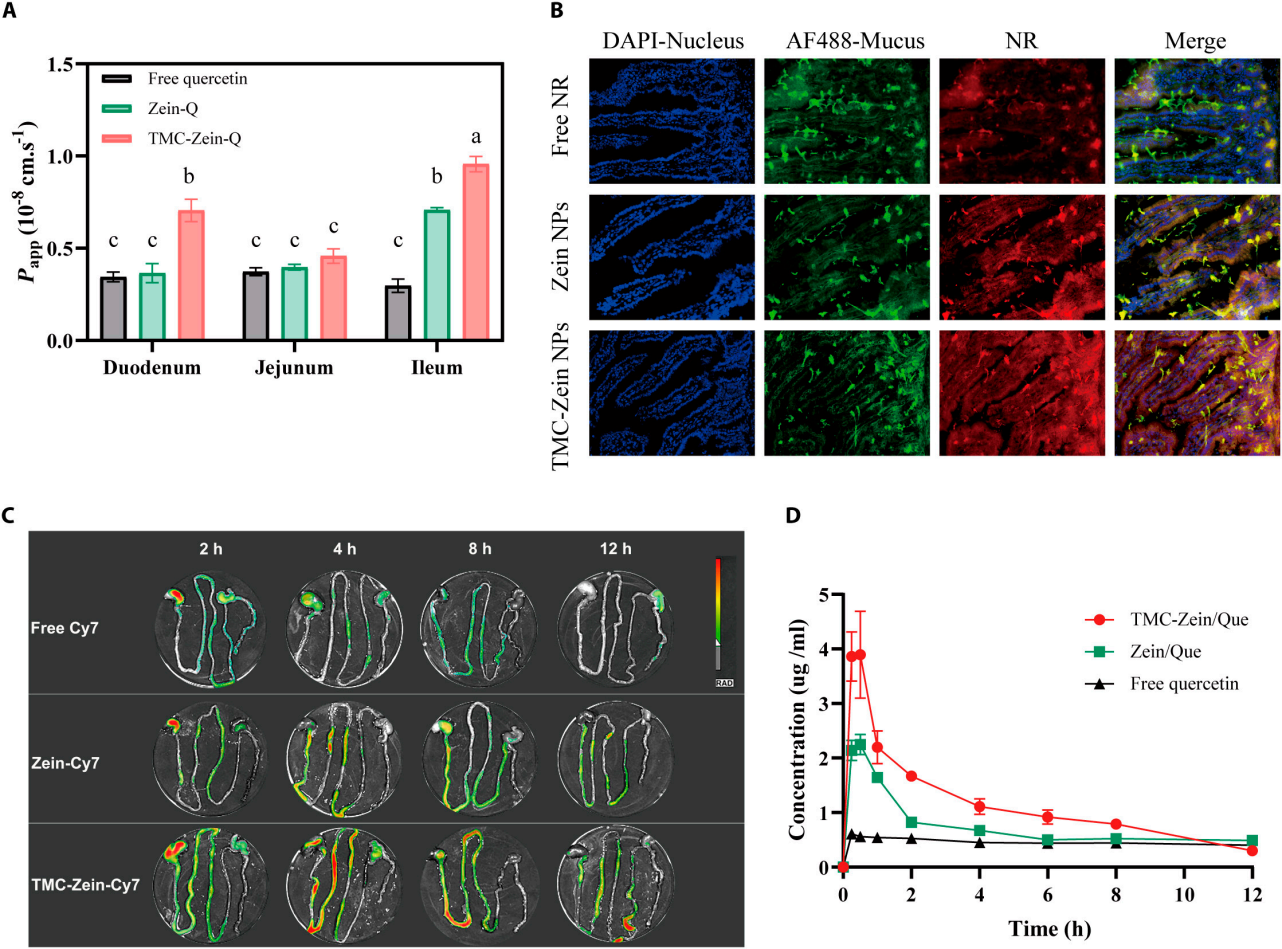
Intestinal permeability, mucoadhesion, and pharmacodynamic study of TMC-Zein-Q. (A) *P*_app_ values of free quercetin, Zein-Q, or TMC-Zein-Q after 2-h incubation in excised intestines. The data are expressed as mean ± SEM (*n* = 3). (B) In vivo absorption of free NR, Zein-NR, or TMC-Zein-NR in small intestinal tissues after 2 h of oral administration. The nuclei of intestinal epithelial cells were labeled with blue fluorescence, and the hydrophilic mucus layer on the surface of small intestinal villi was labeled with green fluorescence. (C) Distribution of free Cy7, Zein-Cy7, and TMC-Zein-Cy7 nanoparticles in gastrointestinal tract after gavage administration over time. (D) Plasma concentration of quercetin after oral administration of free quercetin (50 mg/kg body weight) and TMC- Zein-Q in mice. The data are expressed as mean ± SEM (*n* = 4). Different lowercase letters (a, b, c, d) indicate statistically significant differences between groups (*P* < 0.05). Groups sharing at least one common letter are not significantly different from each other, while groups with completely different letters exhibit significant differences.

To further confirm the enhanced intestinal permeability of quercetin and better mimic the in vivo absorption, free quercetin, Zein-Q, and TMC-Zein-Q across the intestine of mice were evaluated. The quercetin transport rates showed the highest level in TMC-Zein-Q but the lowest if only free quercetin existed (Fig. 4A). In addition, TMC-Zein-Q has the highest permeability in the ileum, which may be related to the sustained-release characteristics of nanoparticles. Therefore, the ileum may be the main absorption site of TMC-Zein-Q bilayer nanoparticles. Moreover, the in situ absorption of quercetin-loaded nanoparticles in the small intestine was visualized by using CLSM to evaluate the enhanced effects. The results showed that a slight signal was detected in microvilli for free NR. In contrast, stronger signals were observed within microvilli for Zein-NR, and yet the strongest signals were observed for the TMC-Zein-NR group within microvilli, indicating the highest absorption of NR (Fig. 4B). These results demonstrated that TMC-Zein-Q indeed exhibited better intestinal permeability than Zein-Q and free quercetin. Combined with the results of the transepithelial transport study of nanoparticles, this intestinal permeability effect may be related to the paracellular pathway, which was reversibly opened by TMC.

The fluorescence intensity of free Cy7 in the gastrointestinal tract decreased rapidly after oral administration, and the fluorescence disappeared completely after 12 h of gavage (Fig. 4C). In contrast, Zein-Cy7 prolonged the retention of Cy7 fluorescence, and as expected, TMC-Zein-Cy7 had the longest fluorescence retention time. After 8 h of intragastric administration, there was a strong fluorescence intensity in the ileum, and low intensity fluorescence was still distributed in the duodenum, jejunum, and ileum after 12 h. The fluorescence intensity of the jejunum was significantly higher than that of Zein-Cy7 at 8 h after intragastric administration. When TMC was used as the shell, the nanoparticles moved more slowly along the intestine, implying that the TMC-based nanoparticles had better mucoadhesive properties. This phenomenon may be due to the electrostatic interaction between the positively charged TMC and the negatively charged sialic acid residues on the mucosal surface [12]. Generally, the mucoadhesive property plays a beneficial role in enhancing the uptake of nanoparticles by epithelial cells [41]. It could provide a prolonged contact between the nanoparticles and the absorptive surface, thus promoting their oral absorption capacity [8].

### In vivo pharmacodynamic study of TMC-Zein-Q core–shell nanoparticles

The results showed that the plasma concentration of quercetin decreased after a brief peak (15 to 30 min), and TMC-Zein-Q effectively improved the plasma concentration of quercetin. The overall pharmacokinetic curve showed a trend of TMC-Zein-Q > Zein-Q > free quercetin (Fig. 4D). As expected, TMC-Zein-Q demonstrated the highest plasma concentration of quercetin (3.89 ± 1.59 μg/ml) when compared with the Zein-Q (2.25 ± 0.37 μg/ml) and free quercetin (0.61 ± 0.06 μg/ml) groups. Moreover, the relative bioavailability of TMC-Zein-Q was increased 2.48-fold when compared with free quercetin. These observations corresponded to the results of intestinal permeability and absorption, suggesting that the enhanced bioavailability of quercetin may be attributable to the permeation-enhancing effect of TMC. In this paper, the peak time of quercetin was at 15 to 30 min, which was similar to previous studies [42]. However, the concentration of quercetin was relatively low after 2 h, which may be due to the rapid metabolism of quercetin [1].

### TMC-Zein-Q core–shell nanoparticles alleviated obesity in HFD-fed mice

**Fig. 5.**
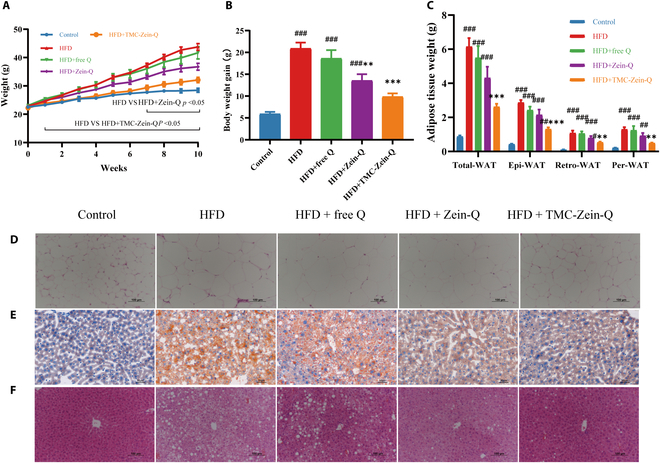
Effects of TMC-Zein-Q on anti-obesity effect of quercetin in mice. (A) Body weight time course measurements. (B) Weight gain and (C) fat mass of mice after 10 weeks of intervention. The data are expressed as mean ± SEM (*n* = 8). (D) H&E staining of epididymal fat sections. (E) Liver Oil Red O staining and (F) H&E staining of liver tissues. *P < 0.05, **P < 0.01, and ***P < 0.001 compared with the HFD group. ^#^*P* < 0.05, ^##^*P* < 0.01, and ^###^*P* < 0.001 compared with the control group.

The anti-obesity effect of TMC-Zein-Q was investigated in mice, and the results showed that the weight of mice in the TMC-Zein-Q group was significantly decreased after 1 week of intervention (*P* < 0.05; Fig. 5A), whereas the mice in the Zein-Q group showed weight loss until 7 weeks of intervention. As expected, after 10 weeks of intervention, TMC-Zein-Q showed the most weight loss effect (*P* < 0.01) (Fig. 5B). Furthermore, the decrease in total-WAT weight (epi-WAT, retro-WAT, and per-WAT) was found in mice with TMC-Zein-Q intervention (Fig. 5C). Histological analysis showed that the size of epididymal adipocytes in the TMC-Zein-Q group was also decreased compared with the HFD group (Fig. 5D). The hepatic steatosis caused by HFD was alleviated in all treatment groups, among which the TMC-Zein-Q-treated group showed the lowest liver lipid accumulation and fat cavitations (Fig. 5E and F).

**Fig. 6.**
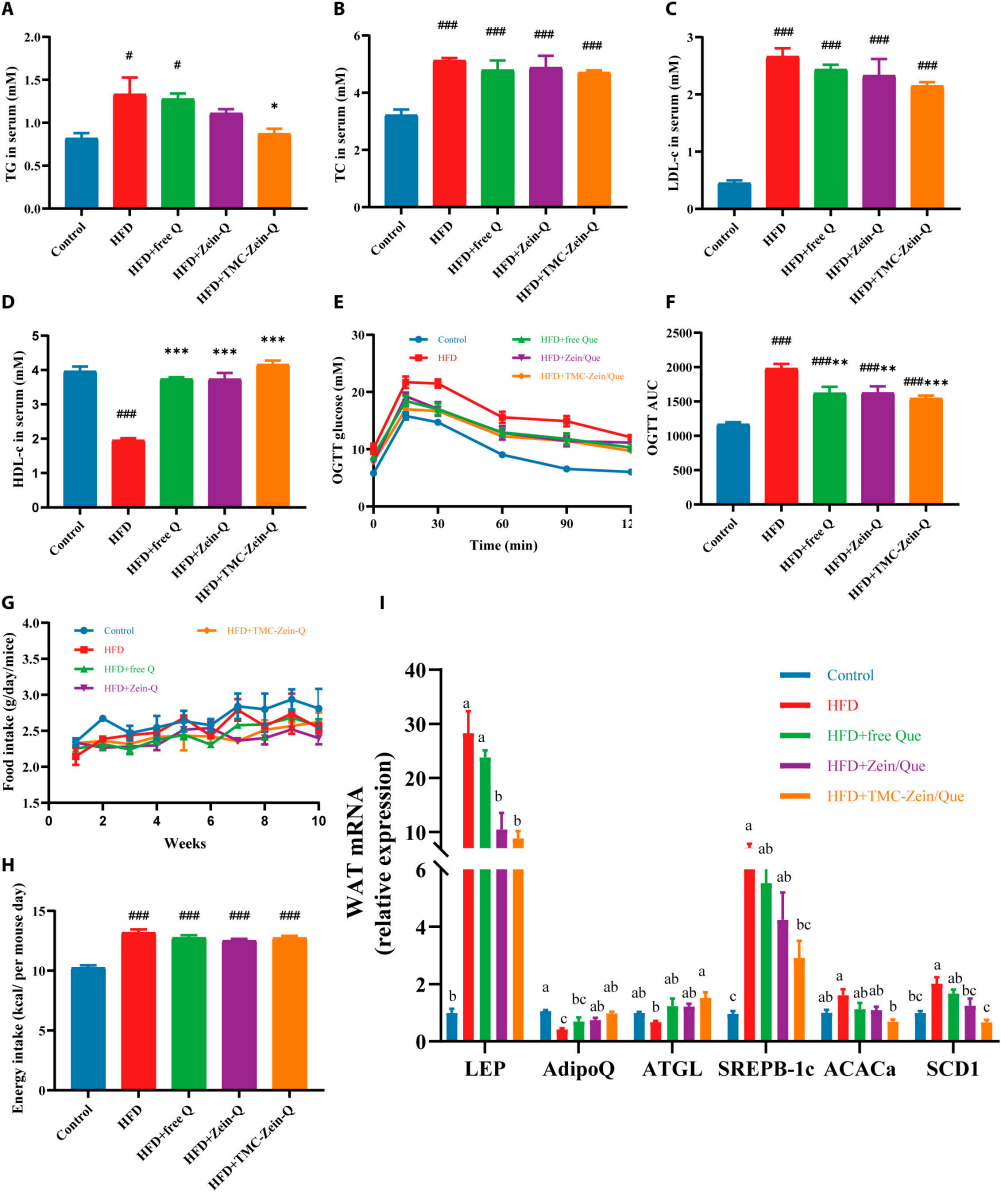
Effects of TMC-Zein-Q on serum lipid profiles, glucose tolerance, and mRNA expression of AMPK-related genes. (A) TG. (B) TC. (C) LDL-c. (D) HDL-c. (E) OGTT. (F) AUC. (G) Food intake and (H) energy intake of mice. The data are expressed as mean ± SEM (*n* = 8). (I) Real-time PCR assay for the differentially expressed genes in the AMPK signaling pathway. The data are expressed as mean ± SEM (*n* = 6). *P < 0.05, **P < 0.01, and ***P < 0.001 compared with the HFD group. ^#^*P* < 0.05, ^##^*P* < 0.01, and ^###^*P* < 0.001 compared with the control group. Different lowercase letters (a, b, c, d) indicate statistically significant differences between groups (*P* < 0.05). Groups sharing at least one common letter are not significantly different from each other, while groups with completely different letters exhibit significant differences.

A growing body of clinical evidence supports the close linkage among obesity, insulin resistance, and dyslipidemia [43,44]. Serum lipids were measured, and the greatest improvements in TG, TC, LDL-c, and HDL-c were found in the TMC-Zein-Q group (Fig. 6A to D). More specifically, treatment with TMC-Zein-Q decreased the TG levels in mice, while this effect was not found in the free Q and Zein-Q groups. Additionally, all intervention groups could significantly reduce the AUC of the OGTT, and TMC-Zein-Q showed the most significant effect (*P* < 0.01) (Fig. 6E and F). Importantly, the food intake of the mice in the TMC-Zein-Q group was not different from that in the HFD group (Fig. 6G and H), indicating that those beneficial effects were not due to diet intake.

The adenosine 5'-monophosphate-activated protein kinase (AMPK) signaling pathway plays an important role in regulating energy balance and lipid metabolism, and the activation of AMPK is one of the important targets in obesity treatment. To further investigate the mechanism of TMC-Zein-Q alleviating obesity, the mRNA expression of AMPK-related upstream and downstream genes was analyzed in epididymal adipocytes. The mRNA expression results showed that all treatments reversed the changes in mRNA levels of the lipogenesis-related genes *LEP*, *AdipoQ*, *ATGL*, *SREBP-1c*, *ACACa*, and *SCD1* in mice. As expected, these reversals were most notable in the TMC-Zein-Q group (Fig. 6I). Adiponectin and leptin could regulate the phosphorylation of AMPK [45], leading to the up-regulation of *ATGL*, the first key enzyme in lipolysis, thereby promoting the process of lipolysis. Lipid synthesis was regulated by the *SREBP-1c* transcription factor [46], and *p-AMPK* could inhibit *SREBP-1c* expression [47], thereby reducing the activity of *SCD1* and *ACACa*.

Previous studies on improving the oral absorption of quercetin generally had no validation in vivo and thus lacked credibility [48,49]. In this study, the oral absorption enhancement effect of TMC-Zein-Q nanoparticles was evaluated in vitro and in vivo, and their anti-obesity effect was verified by animal experiments. Our results demonstrated that TMC-Zein-Q nanoparticles could effectively improve the oral absorption and anti-obesity effect of quercetin, which had a promising application.

### The mechanisms of TMC-Zein-Q core–shell nanoparticles alleviating obesity

**Fig. 7.**
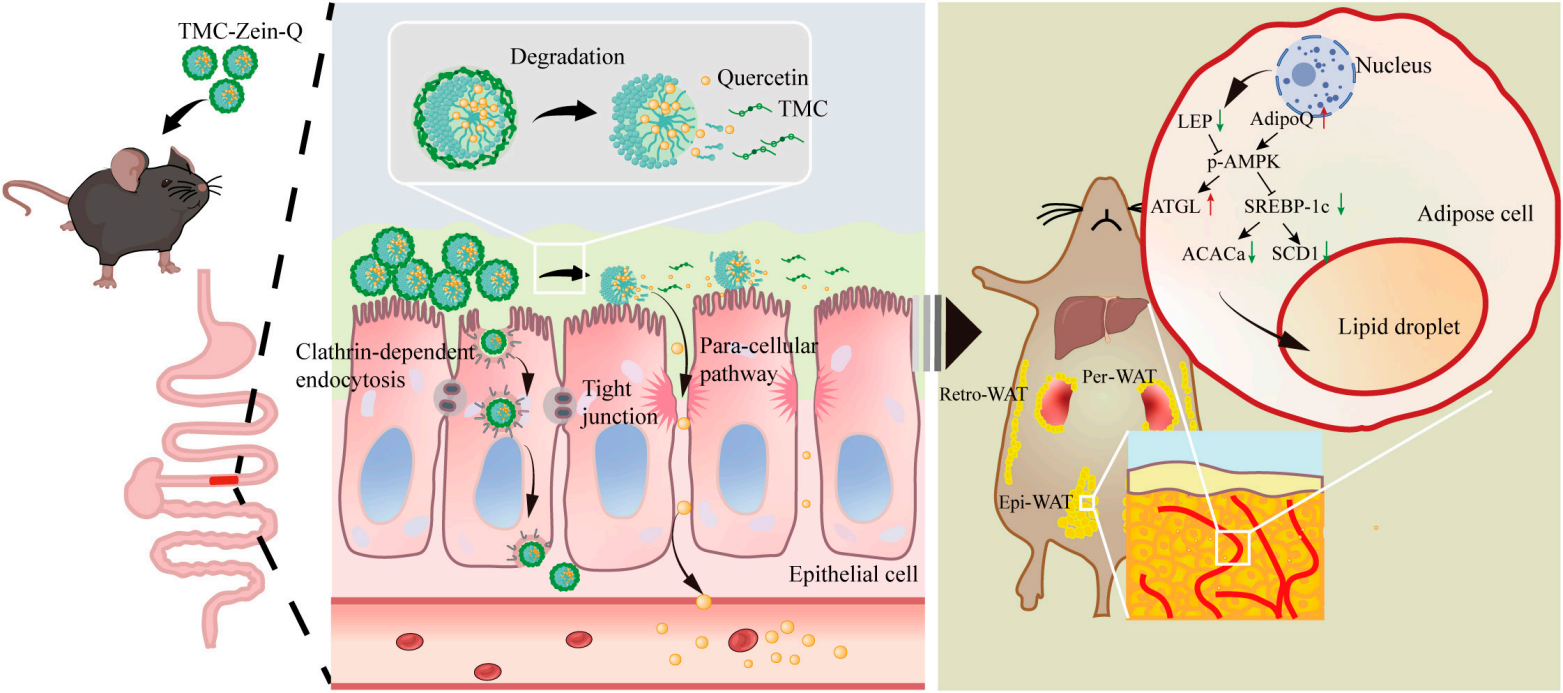
Schematic illustration of TMC-Zein-Q nanoparticles for anti-obesity. TMC-Zein-Q nanoparticles showed great intestinal permeability and mucoadhesion, and improved the bioavailability of quercetin in mice via the paracellular pathway. They could significantly inhibit the weight gain in mice fed with HFD and increase the mRNA expression levels of *AdipoQ* and *ATGL* while significantly decreasing the levels of *LEP*, *SREBP-1c*, *ACACa*, and *SCD1* in adipocytes.

In this study, TMC-Zein-Q core–shell nanoparticles were designed to improve the oral absorption of quercetin (Fig. 7). The nanoparticles consist of quercetin-loaded zein core and TMC shell, which had high EE, DLC, and good solubility. These nanoparticles could protect quercetin from degradation and show the sustained-release effect in an in vitro drug release experiment. In addition, they exhibited good biosafety and were found to reversibly open tight junctions in Caco-2 cells to improve the absorption of quercetin through the paracellular pathway. Furthermore, nanoparticles showed stronger intestinal permeability and mucoadhesion in vivo, thus increasing the blood concentration of quercetin in mice. Additionally, TMC-Zein-Q nanoparticles showed the most weight loss effect in the obese mouse model after 10 weeks of intervention. It could significantly inhibit the weight gain in mice fed with HFD and increase the mRNA expression levels of *AdipoQ* and *ATGL* while significantly decreasing the levels of *LEP*, *SREBP-1c*, *ACACa*, and *SCD1* in adipocytes. Moreover, serum lipid levels, hepatic steatosis, and glucose tolerance were also improved by TMC-Zein-Q nanoparticles. All these beneficial effects might be attributed to the increased intestinal absorption caused by TMC-Zein-Q nanoparticles via the paracellular pathway, thus improving the bioavailability of quercetin.

## Conclusion

In conclusion, we successfully constructed the TMC-Zein-Q core–shell structure for oral quercetin delivery. These nanoparticles possessed ideal gastrointestinal stability and intestinal permeability, which could remarkably increase the anti-obesity effect of quercetin. Our results indicated that TMC-Zein-Q provided a novel and feasible strategy for the treatment of obesity via the oral route. However, the complex physiological environment of the human body may affect the function and biological activity of this delivery system, and the clinical application of nanoparticles still needs further study.

## Data Availability

The data that support the findings of this study are available from the corresponding author upon reasonable request.
