# Supplementary material for: Zein and Trimethyl Chitosan-Based Core–Shell Nanoparticles for Quercetin Oral Delivery to Enhance Absorption by Paracellular Pathway in Obesity Mice
Source: Biomater Res. 2025 Apr 28;29:0193. doi: 10.34133/bmr.0193 (PMC12034925; doi:10.34133/bmr.0193)
Supplement: Supplementary 1 — Table S1 [file bmr.0193.f1.docx]

**Table S1**. Primer sequence

| Gene | Forward primer (5’-3’) | Reverse primer (5’-3’) |
| --- | --- | --- |
| *Gapdh* | GTGTTCCTACCCCCAATGTGT | ATTGTCATACCAGGAAATGAGCTT |
| *Lep* | GAGACCCCTGTGTCGGTTC | CTGCGTGTGTGAAATGTCATTG |
| *Adipoq* | TGTTCCTCTTAATCCTGCCCA | CCAACCTGCACAAGTTCCCTT |
| *Atgl* | AACACCAGCATCCAGTTCAA | GGTTCAGTAGGCCATTCCTC |
| *Srebp-1c* | GATGTGCGAACTGGACACAG | CATAGGGGGCGTCAAACAG |
| *Acca* | CTCCCGATTCATAATTGGGTCTG | TCGACCTTGTTTTACTAGGTGC |
| *Scd1* | CACCTGCCTCTTCGGGATTT | CTTTGACAGCCGGGTGTTTG |
